# Evidence based policy making during times of uncertainty through the lens of future policy makers: four recommendations to harmonise and guide health policy making in the future

**DOI:** 10.1186/s13690-022-00898-z

**Published:** 2022-05-18

**Authors:** Margaux Françoise, Cléa Frambourt, Paige Goodwin, Fabian Haggerty, Marjolaine Jacques, Maya-Lhanze Lama, Clara Leroy, Augustin Martin, Raquel Melgar Calderon, Jean Robert, Elena Schulz-Ruthenberg, Lina Tafur, Mona Nasser, Louisa Stüwe

**Affiliations:** 1 Sciences Po, Paris, France; 2 Peninsula Dental School, Faculty of Health, University of Plymouth, Plymouth, UK

**Keywords:** Health policy, Ethics, Evidence based policy making, Policy recommendations

## Abstract

The Covid-19 pandemic has not only outlined the importance of using evidence in the healthcare policy making process but also the complexity that exists between policymakers and the scientific community. As a matter of fact, scientific data is just one of many other concurrent factors, including economic, social and cultural, that may provide the rationale for policy making. The pandemic has also raised citizens’ awareness and represented an unprecedented moment of willingness to access and understand the evidence underpinning health policies.

This commentary provides policy recommendations to improve evidence-based policy making in health, through the lens of a young generation of public policy students and future policymakers, enrolled in a 24-hour course at Sciences Po Paris entitled “Evidence-based policy-making in health: theory and practice(s)”.

Four out of 11 recommendations were prioritised and presented in this commentary which target both policymakers and the scientific community to make better use of evidence-based policy making in health. First, policy makers and scientists should build trusting partnerships with citizens and engage them, especially those facing our target health care issues or systems. Second, while artificial intelligence raises new opportunities in healthcare, its use in contexts of uncertainty should be addressed by policymakers in terms of liability and ethics. Third, conflicts of interest must be disclosed as much as possible and effectively managed to (re) build a trust relationship between policymakers, the scientific community and citizens, implying the need for risk management tools and cross border disclosure mechanisms. Last, well-designed and secure health information systems need to be implemented, following the FAIR (findable, accessible, interoperable and reusable) principles for health data. This will take us a step further from data to ‘policy wisdom’.

Overall, these recommendations identified and formulated by students highlight some key issues that need to be rethought in the health policy cycle through elements like institutional incentives, cultural changes and dialogue between policy makers and the scientific community. This input from a younger generation of students highlights the importance of making the conversation on evidence-based policy making in health accessible to all generations and backgrounds.

## Background

The Covid-19 pandemic has magnified the complexities of the healthcare policy making process, and in particular, the interactions between policymakers and the scientific community. Despite the importance and impact of health policies on the general public, most people, including the young generation, are not able to access and understand information on the details of the scientific analysis underpinning those policies or the process to translate scientific data to policy making. In this commentary, a group of graduate students identified and discussed some of the key issues to improve the approach to set evidence informed health policies considering the diminishing trust of citizens towards policy organisations due to the current infodemic.

As a matter of fact, the trust of citizens in science and policy making has been heavily impacted in the face of a new “infodemic”, as described by Tedros Adhanom Ghebreyesus [1], the Director General of the World Health Organisation, with the increase of fake news spreading faster and faster, fueling a climate of mistrust regarding both science and policymakers. Moreover, the notion of a ‘social contract’ uniting citizens and the State on the basis of the application of measures promoted by governments has again emerged and is said to be strained in the context of Covid and in particular following numerous waves and virus variants, which have called for constantly modified restrictions [2].

Consequently, the impact of the Covid-19 pandemic on society resulted in more people wanting to understand the scientific analysis and rationale behind the policies especially when there are large variations in how the data is interpreted in different countries and how countries have responded to it. It has shown that scientific evidence is just one of many other concurrent factors, including economic, social, psychological and cultural, that may influence the political decision-making process. The policy making process therefore consists of arbitrations between the desirable, possible and acceptable for society [3].

The production, effective dissemination and use of research in healthcare policy making is hence a complex and multifaceted process but above all a means to strengthen interactions and mutual understanding not only between policymakers and the scientific community but also between these stakeholders and the general population.

This commentary provides policy recommendations to improve evidence-based policy making in health, through the lens of a young generation of public policy students, which target both policymakers and the scientific community.

## Methods

These initial observations coupled with introductory content on the main definitions, concepts and stakeholders of evidence-based policy making (EBPM) in health were the starting point for a A group of 12 graduate students from diverse academic backgrounds including biostatistics, political science, law, economy, sociology and gender studies, got acquainted with definitions, concepts and stakeholders of EBPM in health through a 24-hour course at Sciences Po Paris entitled “Evidence-based policy-making in health: theory and practice(s)”.

In groups of two, students developed a total of 11 policy recommendations, built on presentations provided by renowned thematic experts during the course, literature review and grey literature search as well as their personal experiences. All recommendations were cross checked for quality, coherence and references by other pairs of students. The resulting set of recommendations provide a totally new lense to EBPM in health as they emerge from a very young generation of students born in the late 1990s with the majority having just started first professional experiences in the policy field. Their recommendations, valid both at the international and national level, are targeted both at policymakers and the scientific community to work together towards a more qualitative approach to knowledge co-creation and more effective policy-practice feedback loops, as well as a clearer definition of all steps of health research agendas and uptake of results into policy. Students prioritised four out of eleven recommendations, highlighting the key elements to work for improved EBPM in health, which are presented here.

## Results

First, rebuilding trust in health policy making amongst citizens should be a priority. Citizens should be placed at the core of EBPM through the reinforcement of their mandate as actors of innovation. For example, this can be achieved through the institutionalisation of data altruism practices, making their data available for secondary use purposes while remaining under their control, in the respect of the General Data Protection Regulation (GDPR), security and privacy concerns.

Further, in the framework of health democracy, citizens become directly involved in the design and adoption of national health priorities as proven in the context of the revision of the French bioethics law, where a public consultation has been conducted involving patient associations and advocacy organisations [4]. Such practices should be generalised, especially regarding ethics-related issues, despite being resource-intensive. They allow citizens to be represented in the democratic public debate and the policy making process to make their needs heard, including the complexity of patient experiences, not always known by policymakers. Representation can also include and sensitise the wider population through online or workshop consultations, juries and open democracy discussions in order to better inform stakeholders about ongoing initiatives and have them express their views. For example, public consultations are a common tool in the preparation of all major European Union (EU)-level directives and regulations.

While the outcomes of such public opinions may not be unconditionally binding for policymakers, consultations and the justification of having considered citizen views should be mandatory in the health decision-making process. Nonetheless, achieving citizen involvement takes time and effort is necessary to convince citizens of the value of their involvement and encourage them to take part in it. The institutionalization of citizen involvement therefore needs to come along with the building of a culture of trust both in science and policy making. This is already part of the DNA of political systems in some countries from lowest school age (i.e. Nordic countries like Denmark), ensuring that citizen’s input is obtained, visible, transparently considered and used.

On this basis, EBPM, if employed fully, may strengthen the trust of citizens and their adherence to health policies. Citizens themselves are increasingly considered ‘actors of innovation’ as providers of evidence themselves, i.e. through participation in clinical trials or active data sharing. For example, evidence clearly and consistently shows that rare disease patients, regardless of the severity of their disease and their socio-demographic profile, are supportive of data sharing to foster research and improve healthcare [5] which may result in better policy making.

Secondly, citizens’ trust in science and policymakers needs to be ensured not only by an increased involvement in the decision making process but also by a strong and transparent regulation regarding EBPM tools. Indeed, the development of artificial intelligence (AI) driven healthcare has raised new opportunities and challenges to the healthcare policy and decision-making process. While AI has enabled healthcare actors to save time and provide more targeted and effective interventions to patients worldwide, evidence is still limited on their long-term implications, but from an organisational, financial and public health perspective, many health technologies are still under the process of development. The adoption of AI-based software in healthcare and digital therapeutics (DTx) have recently raised the question of liability [6]. In a context in which algorithms keep learning, there is still no consensus regarding the liability doctrine for DTx as there is for the adoption of medicines and medical devices. In order for AI to be a relevant tool to support decision-making in the context of uncertainty and evolving science, its development needs to be accompanied by incentives for policymakers and public sector officials to partake in evidence-based policy making and ethics courses. These could be provided nationally but also internationally, with guidance from the newly established WHO Academy which could include EBPM as one of its priorities.

Overall, data is a crucial element to research algorithms understanding and evaluation but is not always made available to policymakers. Therefore, health data accessibility needs to be increased and the current lack of transparency in data management processes addressed through the promotion of open-source solutions, allowing more transparency of how algorithms are developed. These recommendations apply both to the public and the private sector.

Third, transparency should not only concern data management processes but conflicts of interest (COI) in health as well. COI may influence outcomes in medical practice, education and research, which constitute the main material for policymakers in the decision-making process. They may also directly bias policy making, when policymakers are directly influenced as it is still a widespread practice with 72% of the guidelines issued in the US being elaborated with members of committees in charge of these guidelines having financial ties with the pharmaceutical and device industries for example [7]. Addressing COI is a priority in guaranteeing patients and society’s trust in research and science in general.

Effectively managing COIs relies on the implementation of ban and restriction measures surrounding the participation of decision-making bodies depending on circumstances assessed through a risk-management approach. Potential conflicts of interest should not be damned per se as they are part of every professional’s life, but their existence should be made more transparent through the development of international standards and the generalisation of disclosures as promoted by a new initiatives, such as “Euro for Docs”, that should be reproduced and implemented widely. Overall, governments both at the national and international level, including Horizon funding programmes for European countries, should massively increase funding for patient-centered research programmes to enable research free of any vested interests, or conflicts of interest.

Finally, these recommendations can only be achieved if there are strong health information systems in place. Moving from data to policy wisdom and better informed policy should therefore be our key guiding principle and a concrete translation of EBPM in health.

FAIR (findable, accessible, interoperable and reusable) health data principles, initiatives, implementation practices, and lessons learned in the FAIRification process can meaningfully support both evidence based clinical practice and research transparency [8]. Data driven policy relies on the creation of universal guidelines for the architecture of strong health information systems which should include citizen associations in their governance structure, as is the case of the French Health Data Hub [9] at the national level or sought by regional projects, such as TEHDAS (Towards European Health Data Space). Overall, the development of strong health information systems needs to be an objective shared by all stakeholders across borders to improve EBPM in health.

## Conclusion

A wider development and widespread use of EBPM in health policies could lead to increased trust of citizens in science and health policies. Involving citizens in initiatives that allow them to take part in the policy making process alongside with scientists and policy makers, could improve their understanding and support of using evidence for policy making in health. Such a momentum should be accompanied by measures ensuring the transparent, ethical and innovative use of data ranging from its collection, to the management, exploitation, analysis and use in policies.

The policy recommendations highlight the need to rethink the health policy making cycle, to adopt a more critical eye of how health policy and decisions are being made and what type of evidence is being mobilised. Institutional incentives promoting evidence supply for academics for example need to be accompanied by political incentives to lead evidence-based policies. Nevertheless, for evidence-based policies to be implemented, incentives need to be accompanied by a mindset change among policymakers who should increasingly seek evidence-based health knowledge whilst understanding that doubt and uncertainty are part of science and that scientific evidence takes time as well as for scientists to seek intelligibility and usability for the political process. This mutual understanding of all the stakeholders of the policy making process has to be fostered by trust-reinstauring elements such as the wider inclusion of citizens in the decision-making process and a larger transparency regarding conflicts of interests [10]. EBPM in health cannot exist without strong evidence conveyed by strong health information systems allowing a shift from data to wisdom.

Through these recommendations emerging with input from a younger generation of students, we also intend to demonstrate the importance and relevance of engaging individuals from different backgrounds, nationalities and age groups, specifically the younger generation in EBPM. We hypothesize that this in the long-term also increases engagement and participation in clinical research. As a perspective, we would like to highlight the importance of making the conversation on EBPM accessible not only to senior experts but also to the next generation, and the importance of taking onboard their suggestions for the policies of tomorrow.

## Data Availability

Not applicable.
